# Supplementary material for: Automated Extraction of Multicomponent Alloy Data Using Large Language Models for Sustainable Design
Source: Adv Sci (Weinh). 2026 Jun 9:e75916. Online ahead of print. doi: 10.1002/advs.75916 (PMC13336656; doi:10.1002/advs.75916)
Supplement: Supplementary file 1 — Supporting File: advs75916‐sup‐0001‐SuppMat.pdf. [file ADVS-9999-e75916-s001.pdf]

Supplementary Information on Automated Extraction of Multicomponent Alloy Data Using Large Language Models for Sustainable Design

Aravindan Kamatchi Sundaram[1], Mohit Chakraborty[1], Sai Mani Kumar Devathi[1], B. Pabitramohan Prusty[1], and Rohit Batra[1,2,*]

[1]Department of Metallurgical and Materials Engineering, Indian Institute of Technology Madras, Chennai 600036, India
[2]Center for Atomistic Modelling and Materials Design, IIT Madras, Chennai 600036, India
[*]Author to whom correspondence should be addressed.

March 24, 2026

1 Ablations studies with different LLM models and settings

For our experiments, three OpenAI language models: `GPT-3.5 turbo`, `GPT-4o`, and `GPT-4o mini`, were extensively evaluated on a curated set of fifteen research articles. Model performance was examined under different configurations, including with and without retrieval-augmented generation (RAG). We observed that incorporating RAG consistently improved the performance of the larger models, `GPT-4o` and `GPT-4o mini`. Additionally, an ablation was conducted on response confirmation, wherein an auxiliary LLM call was used to verify the extracted information. Data were retained only when the verification prompt returned an affirmative response. Interestingly, this confirmation step yielded limited benefits for the larger models but was more advantageous for the smaller ones. Overall, both `GPT-4o` and `GPT-4o mini` outperformed `GPT-3.5 turbo` by a significant margin. Considering performance and cost trade-offs, `GPT-4o mini` was employed for smaller text and table extractions, while `GPT-4o` was used for larger and more complex inputs. In both cases, RAG was enabled, and no confirmation step was applied. The F1-scores of the results of this study are reported in Table S1.

Table S1: Model comparison results using F1-scores for different materials science data categories. Various settings were tested including the use of RAG and a second LLM call for confirmation.

**Data category**	**GPT-3.5 turbo**	**GPT-3.5 turbo with confirmation**	**GPT-3.5 turbo with RAG and confirmation**	**GPT-4o with RAG and confirmation**	**GPT-4o with RAG and without confirmation**	**GPT-4o mini with RAG and without confirmation**
Alloys	0.70	0.90	0.87	0.92	0.96	0.92
Processing Conditions	0.80	0.87	0.84	0.91	0.96	0.81
Characterization techniques	0.63	0.88	0.83	0.82	0.99	0.93
Properties	0.86	0.83	0.89	0.75	0.89	0.94

2 Database 1 (DB1) statistics

DB1 contained a total of 37,711 alloy records. Among all the articles considered for DB1 construction, 3,367 (31%) discussed more than one alloy system with the same nominal composition, typically reflecting variations in processing conditions within a single study. For example, Al_3Ti may be reported in as-cast or splat-quenched forms, subjected to different heat-treatment cycles, or mechanically processed through varying degrees of cold rolling. In

addition, 2,202 alloy compositions appeared in more than one article, usually because they were studied under different processing conditions or studied for different properties. As shown in Figure S1, approximately 1,200 alloy compositions were reported in two different articles, about 350 in three articles, and fewer in larger numbers of studies. Figure S1b and c respectively show the elemental and elemental pair distribution in the database. Word cloud plots visualizing the top reported properties and characterization techniques are included in Figure S1d and e, respectively.

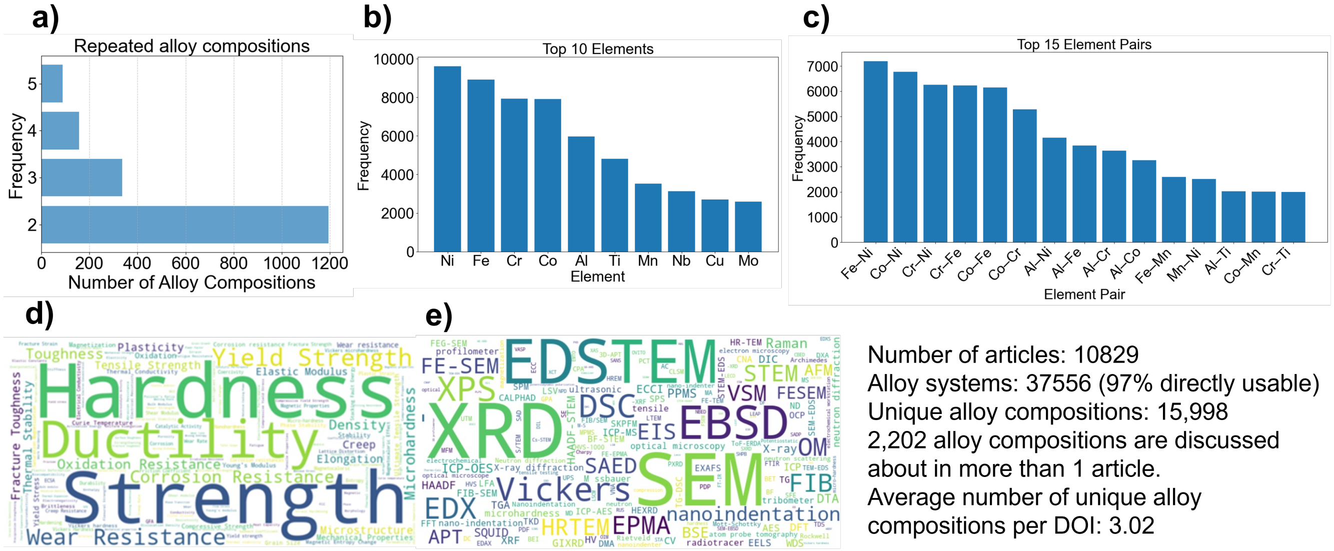

Figure S1: Statistics of DB1 including the number of articles in which a specific alloy composition was reported, distribution of top frequently occurring elements and elemental pairs, and word cloud plots for extracted property and characterization techniques.

3 Cost, time, and other statistics for LLM-based extraction

Table S2 provide details on the overall cost and time incurred for extraction of datasets 1 and 2. We also provide statistics on number of paragraphs/tables processed by two different LLMs and the associated responses, where ‘True’ and ‘Null’ respectively denote scenarios with a valid or “-" response. In this work, both `GPT-4o` and `GPT-4o mini` were used based on the complexity of the text or table being processed. For QS1, `GPT-4o` was used for challenging articles during alloy composition extraction, identified based on the presence of a subset of manually-flagged few-shot examples during RAG. For QS2, `GPT-4o` was used for the first LLM call and for table larger than 30 cells in the second LLM call. `GPT-4o mini` was used otherwise.

Table S2: Cost, time, and extraction statistics for QS1 and QS2.

	**GPT-4o**	**GPT-4o Mini**
Total Cost	∼$312	∼$23
**Q1: Text extraction**		
Cost (USD)	$141.94	$17.92
Paragraphs processed	16,854	39,247
True entries (total)	114,245	113,459
Null entries (total)	313,631	347,399
– Alloy (True / Null)	8,073 / 8,781	11,205 / 28,042
– Processing (True / Null)	26,791 / 110,883	25,516 / 115,021
– Characterization (True / Null)	33,960 / 103,714	32,513 / 108,024
– Properties (True / Null)	46,421 / 91,253	44,225 / 96,312
Total Time (sec)	831,469.43	
Total Time (hrs)	230.96	
**Q2: Table Extraction**		
Cost (USD, approx.)	∼$170	∼$5
Tables processed	12,589	9,808
Successful tables	9,230	4,771
Null tables	3,359	5,037
Total Time (sec)	151,679.58	
Total Time (hrs)	42.13	

4 Distribution of errors made by the LLM

Table S3: Summary of extraction errors across evaluation of QS1 and QS2 for review article dataset.

**Error Type**	**Amount of Error**	**Percentage of Cases**
Composition parsing errors for QS1	49 out of 244 extracted entries	20%
Composition parsing errors for QS2	28 out of 1514 extracted entries	2%
Missing due to complex property descriptions for QS2	321 out of 1847 relevant entries	17.3%
Formatting errors for QS2	12 out of 1514 extracted entries	0.7%
Incorrect association errors for QS2	2 out of 1514 extracted entries	0.13%
Unit interpretation errors for QS2	2 out of 1514 extracted entries	0.13%

In the evaluation of QS1 and QS2, there were various classes of errors made by the LLM which were discussed in section 2.2 and 2.3 of the main manuscript. Here, the specific breakdown and details of the various error types are provided. To understand the types of errors, the counts are on the basis of the review article evaluation for QS1 and the second review article for QS2. It was found in QS1 that 20% of all extracted entries had composition parsing errors whereas in QS2 this number dropped to around 2% of the cases. This could be attributed to increased complexity of reporting compositions in textual sections of the paper as compared to how they are reported in tables. 10% of the composition parsing errors for QS1 were random in nature and could not be attributed to any logical or arithmetic error. It was also observed that the LLM misses nearly 17.3% of all available entries due to complex methods of reporting properties. One thing in common for both of the above is that, any extraction that may require multi-step reasoning causes a significant drop in model performance. Additionally, there were no errors that were random in nature in QS2 and all the errors could be categorized into the 5 main categories listed in Table 4. Incorrect association errors refers to those errors where the cells in a table are incorrectly interpreted by the LLM. The pair of incorrect association errors observed occurred in a relatively large-sized table where the LLM exchanged the property values between 2 alloys. Unit interpretation errors refers to the incorrect extraction of the units and occurred in a table where the properties were reported in the scientific notation format (e.g., x*1e-y units and the LLM missed the 1e-y in the column header). Again, this could be attributed to multi-step reasoning as well.

Table 4 contains the classification of the error types for the random subset of papers taken from the target set of articles. Comparing the percentages of the various errors across the too evaluations, the similarities in errors are evident. For QS1, there were 165 relevant entries in the 30 papers. The LLM extracted 146 entries out of which

Table S4: Summary of extraction errors across evaluation of QS1 and QS2 for random 30 articles dataset.

**Error Type**	**Amount of Error**	**Percentage of Cases**
Composition parsing errors for QS1	29 out of 146 extracted entries	19.7%
Composition parsing errors for QS2	6 out of 640 extracted entries	0.9%
Missing due to complex property descriptions for QS2	220 out of 854 relevant entries	25%

117 were extracted exactly as present in the papers. 29 entries were composition parsing errors. This contributed to a precision of 0.8 and a recall of 0.7. In QS2, there were 854 relevant entries out of which 634 matched exactly with the LLM extracted entries. There were 6 entries which had incorrect compositions in the LLM extraction. This contributed to a precision of 0.99 and a recall of 0.75.

5 Modified RAG for context retrieval in QS2

Preliminary experiments using RAG for context retrieval in QS2 revealed that the presence of symbols, subscripts and superscripts (e.g., σ_y, μ_s) often degraded retrieval performance. This could be because the embedding space is not optimized to handle non-ASCII characters. Thus, to improve the RAG performance, non-ASCII symbols were converted to ASCII representations (e.g., σ to sigma), while subscripts and superscripts were prepended with '_' and '^', respectively. This was observed to significantly improve the retrieval efficiency, making the context creation much more accurate.

6 Potential sustainable candidates identified using DB2

Table S5: List of promising candidates identified in the domain of soft magnets with high sustainability index. Relevant properties and source article information for each candidate is also included. $\mathbf{M_s}$: saturation magnetization, $\mathbf{H_c}$: coercivity, $\mathbf{E_{spec}}$: specific magnetic energy capacity.

**Alloy / Material**	**Sustainability index**	**Property**	**Value**	**Unit**	**DOI / Source**
**High-Performance Magnetic Candidates**					
$FeCoNi(AlMn)_{0.1}$	0.839	$\mathbf{M_s}$	145.8	$A \cdot m^2/kg$	10.1016/j.jallcom.2022.164724
		$\mathbf{H_c}$	116.8	A/m	10.1016/j.mattod.2021.03.018
		$\mathbf{E_{spec}}$	5.35×10^{-6}	kJ/kg	10.1016/j.pmatsci.2024.101332
$Al_{17.6}Co_{16.5}Cr_{17.2}Fe_{17.1}Ni_{17.9}Si_{13.7}$	0.839	$\mathbf{M_s}$	37.1	$A \cdot m^2/kg$	10.1016/j.jallcom.2022.164074
		$\mathbf{H_c}$	3.6	A/m	
		$\mathbf{E_{spec}}$	4.26×10^{-8}	kJ/kg	
$Al_{19.0}Co_{18.6}Cr_{19.1}Fe_{19.3}Ni_{19.1}Si_{4.9}$	0.838	$\mathbf{M_s}$	45.4	$A \cdot m^2/kg$	10.1016/j.jallcom.2022.164074
		$\mathbf{H_c}$	2.4	A/m	
		$\mathbf{E_{spec}}$	3.43×10^{-8}	kJ/kg	
$Al_{17.5}Co_{18.3}Cr_{18.5}Fe_{18.3}Ni_{18.0}Si_{9.4}$	0.837	$\mathbf{M_s}$	45.7	$A \cdot m^2/kg$	10.1016/j.jallcom.2022.164074
		$\mathbf{H_c}$	4.4	A/m	
		$\mathbf{E_{spec}}$	6.32×10^{-8}	kJ/kg	
$Fe_{35}Co_{35}Mn_{9}Al_{21}$	0.835	$\mathbf{M_s}$	142.2	$A \cdot m^2/kg$	10.1016/j.matdes.2024.112787
		$\mathbf{H_c}$	144.9	A/m	
		$\mathbf{E_{spec}}$	6.47×10^{-6}	kJ/kg	
$Fe_{35}Co_{35}Mn_{15}Al_{15}$	0.832	$\mathbf{M_s}$	162.8	$A \cdot m^2/kg$	10.1016/j.matdes.2024.112787
		$\mathbf{H_c}$	167.2	A/m	
		$\mathbf{E_{spec}}$	8.55×10^{-6}	kJ/kg	
$Al_{3}Co_{7}Fe_{7}$	0.831	$\mathbf{M_s}$	157.7	$A \cdot m^2/kg$	10.1016/j.actamat.2024.119686
		$\mathbf{H_c}$	170.8	A/m	
		$\mathbf{E_{spec}}$	8.46×10^{-6}	kJ/kg	
$CoFeMn_{0.5}Al_{0.25}$	0.830	$\mathbf{M_s}$	173.0	$A \cdot m^2/kg$	10.1016/j.matchemphys.2023.127518
		$\mathbf{H_c}$	99.5	A/m	
		$\mathbf{E_{spec}}$	5.41×10^{-6}	kJ/kg	
**Flagged Alloys (High Performance, Missing Sustainability Data)**					
$Fe_{24.75}Co_{24.75}Ni_{24.75}(Si_{0.3}B_{0.7})_{24.75}Al_{1}$	-	$\mathbf{M_s}$	96.5	$A \cdot m^2/kg$	10.1016/j.intermet.2024.108304
		$\mathbf{H_c}$	3.2	A/m	
		$\mathbf{E_{spec}}$	9.70×10^{-8}	kJ/kg	
$Fe_{23.75}Co_{23.75}Ni_{23.75}(Si_{0.3}B_{0.7})_{23.75}Al_{5}$	-	$\mathbf{M_s}$	75.7	$A \cdot m^2/kg$	10.1016/j.intermet.2024.108304
		$\mathbf{H_c}$	7.0	A/m	
		$\mathbf{E_{spec}}$	1.66×10^{-7}	kJ/kg	
$Fe_{23}Co_{23}Ni_{23}(Si_{0.3}B_{0.7})_{23}Al_{8}$	-	$\mathbf{M_s}$	63.3	$A \cdot m^2/kg$	10.1016/j.intermet.2024.108304
		$\mathbf{H_c}$	6.6	A/m	
		$\mathbf{E_{spec}}$	1.31×10^{-7}	kJ/kg	
**Industrial Benchmarks**					
Silicon Steel	0.910	$\mathbf{M_s}$	189.0	$A \cdot m^2/kg$	10.1016/j.jmmm.2020.167702
		$\mathbf{H_c}$	< 45.0	A/m	cds.cern.ch/record/181486
		$\mathbf{E_{spec}}$	1.98×10^{-5}	kJ/kg	10.3390/app10186515
Permalloy 80	0.852	$\mathbf{M_s}$	≈ 80.0	$A \cdot m^2/kg$	10.1016/J.JMMM.2009.08.004
		$\mathbf{H_c}$	1.59 - 3.18	A/m	10.2514/6.2004-5749
		$\mathbf{E_{spec}}$	$(2.5–10) \times 10^{-7}$	kJ/kg	ISBN: 978-0-12-269951-1
FINEMET	0.825	$\mathbf{M_s}$	131.3	$A \cdot m^2/kg$	10.1016/S0304-8853(00)00189-X
		$\mathbf{H_c}$	8.0	A/m	10.1016/S0304-8853(00)00189-X
		$\mathbf{E_{spec}}$	4.45×10^{-7}	kJ/kg	10.1016/j.pnsc.2017.09.002
Fe-Co (Hiperco)	0.822	$\mathbf{M_s}$	235.0	$A \cdot m^2/kg$	10.1016/j.jallcom.2020.157998
		$\mathbf{H_c}$	159.0	A/m	10.1063/1.1453939
		$\mathbf{E_{spec}}$	1.11×10^{-5}	kJ/kg	

Table S6: List of promising candidates identified in the domain of lightweighting with high sustainability index. Relevant properties and source article information for each candidate is also included. $\sigma_{\mathbf{y}}$: yield strength, ρ: density, $\sigma_{\mathbf{spec}}$: specific strength (σ_y/ρ).

Alloy / Material	Sustainability index	Property	Value	Unit	DOI / Source
**High-Performance Candidates (Sustainable & Strong)**					
$Fe_{30}(NiMnCuAlTi)_{70}$	0.91	$\sigma_{\mathbf{y}}$	2016.0	MPa	10.1016/j.jallcom.2023.172497
		ρ	6.50	g/cm^3	
		$\sigma_{\mathbf{spec}}$	310.2	$kN \cdot m/kg$	
$Fe_{70}(NiMnCuAlTi)_{30}$	0.91	$\sigma_{\mathbf{y}}$	2046.0	MPa	10.1016/j.jallcom.2023.172497
		ρ	7.25	g/cm^3	
		$\sigma_{\mathbf{spec}}$	282.2	$kN \cdot m/kg$	
AlNbTiZr	0.87	$\sigma_{\mathbf{y}}$	1579.0	MPa	10.1016/j.jmrt.2023.08.149
		ρ	5.85	g/cm^3	10.1016/j.jmst.2022.11.054
		$\sigma_{\mathbf{spec}}$	269.9	$kN \cdot m/kg$	
$CrFeNiAl_{0.3}Ti_{0.3}$	0.87	$\sigma_{\mathbf{y}}$	1712.0	MPa	10.1016/j.msea.2019.138566
		ρ	6.93	g/cm^3	
		$\sigma_{\mathbf{spec}}$	247.1	$kN \cdot m/kg$	
FeNiMnCuAlTi	0.87	$\sigma_{\mathbf{y}}$	1530.0	MPa	10.1016/j.jallcom.2023.172497
		ρ	6.28	g/cm^3	
		$\sigma_{\mathbf{spec}}$	243.6	$kN \cdot m/kg$	
$Ni_{30}(FeMnCuAlTi)_{70}$	0.85	$\sigma_{\mathbf{y}}$	1526.0	MPa	10.1016/j.jallcom.2023.172497
		ρ	6.58	g/cm^3	
		$\sigma_{\mathbf{spec}}$	231.9	$kN \cdot m/kg$	
$Al_{11.7}Cr_{11.7}Nb_{11.7}Ti_{65.0}$	0.88	$\sigma_{\mathbf{y}}$	1128.1	MPa	10.1016/j.intermet.2022.107470
		ρ	5.04	g/cm^3	
		$\sigma_{\mathbf{spec}}$	224.1	$kN \cdot m/kg$	
$Fe_{50}(NiMnCuAlTi)_{50}$	0.91	$\sigma_{\mathbf{y}}$	1525.0	MPa	10.1016/j.jallcom.2023.172497
		ρ	6.86	g/cm^3	
		$\sigma_{\mathbf{spec}}$	222.1	$kN \cdot m/kg$	
**Flagged Alloys (High Strength, Incomplete Sustainability Data)**					
$Al_{20}Li_{20}Mg_{10}Sc_{20}Ti_{30}$	N/A	$\sigma_{\mathbf{y}}$	1970.0	MPa	10.1016/j.mattod.2015.11.026
(Missing: Li, Mg, Sc)		ρ	2.67	g/cm^3	10.1016/j.jallcom.2021.163508
		$\sigma_{\mathbf{spec}}$	737.8	$kN \cdot m/kg$	
$Ti_{50}Zr_{25}Cu_{17}S_8$	N/A	$\sigma_{\mathbf{y}}$	2900.0	MPa	10.1016/j.dt.2023.11.002
(Missing: S)		ρ	5.60	g/cm^3	
		$\sigma_{\mathbf{spec}}$	517.9	$kN \cdot m/kg$	
$(TiZrBeAlCu)_{94}Co_6$	N/A	$\sigma_{\mathbf{y}}$	2055.0	MPa	10.1016/j.jmrt.2023.02.102
(Missing: Be)		ρ	5.27	g/cm^3	
		$\sigma_{\mathbf{spec}}$	389.9	$kN \cdot m/kg$	
$Al_{0.1}CrMoNbVB_{0.015}$	N/A	$\sigma_{\mathbf{y}}$	2933.0	MPa	10.1016/j.jsamd.2024.100688
(Missing: B)		ρ	7.97	g/cm^3	
		$\sigma_{\mathbf{spec}}$	368.0	$kN \cdot m/kg$	
**Industrial Benchmarks**					
AA 7068	0.86	$\sigma_{\mathbf{y}}$	590.0	MPa	10.1088/1757-899X/1059/1/012034
		ρ	2.85	g/cm^3	10.1088/1757-899X/1059/1/012034
		$\sigma_{\mathbf{spec}}$	207.02	$kN \cdot m/kg$	
AA 2195	0.87	$\sigma_{\mathbf{y}}$	550.0	MPa	10.4028/www.scientific.net/MSF.710.125
		ρ	2.71	g/cm^3	10.1063/5.0114421
		$\sigma_{\mathbf{spec}}$	203.0	$kN \cdot m/kg$	
Ti-6Al-4V	0.88	$\sigma_{\mathbf{y}}$	927.0	MPa	10.1063/1.5091886
		ρ	4.43	g/cm^3	10.1016/j.finel.2023.103971
		$\sigma_{\mathbf{spec}}$	209.3	$kN \cdot m/kg$	
AA 2050	0.87	$\sigma_{\mathbf{y}}$	500.0	MPa	ntrs.nasa.gov/citations/20160010564
		ρ	2.70	g/cm^3	10.1007/s11665-009-9554-z
		$\sigma_{\mathbf{spec}}$	185.2	$kN \cdot m/kg$	
AA 7075	0.87	$\sigma_{\mathbf{y}}$	503.0	MPa	10.1016/j.matdes.2010.06.029
		ρ	2.83	g/cm^3	10.1007/s40032-022-00909-6
		$\sigma_{\mathbf{spec}}$	178.0	$kN \cdot m/kg$	
AA 5083	0.87	$\sigma_{\mathbf{y}}$	170.0	MPa	10.3390/ma14185261
		ρ	2.66	g/cm^3	10.3390/ma14185261
		$\sigma_{\mathbf{spec}}$	63.9	$kN \cdot m/kg$	
AA 5052	0.88	$\sigma_{\mathbf{y}}$	89.4	MPa	10.3390/met10050564
		ρ	2.68	g/cm^3	10.1016/j.matpr.2021.10.331
		$\sigma_{\mathbf{spec}}$	33.4	$kN \cdot m/kg$	

Table S7: List of promising candidates identified in the domain of corrosion resistance with high sustainability index. Relevant properties and source article information for each candidate is also included. $\mathbf{I_{corr}}$: corrosion current density (mA/cm^2).

Alloy / Material	Sustainability index	Property	Value	Unit	DOI / Source
**High-Performance Candidates (Sustainable & Corrosion Resistant)**					
$Fe_{0.27}Ni_{0.25}Cr_{0.24}Al_{0.24}$	0.87	$\mathbf{I_{corr}}$<br>$\log(\mathbf{I_{corr}})$	9.26×10^{-6}<br>-5.03	mA/cm^2<br>-	10.1016/j.corsci.2023.111736
$Fe_{0.61}Cr_{0.18}Ni_{0.13}Al_{0.08}$	0.89	$\mathbf{I_{corr}}$<br>$\log(\mathbf{I_{corr}})$	1.60×10^{-4}<br>-3.80	mA/cm^2<br>-	10.1016/j.corsci.2023.111302<br>10.1016/j.corsci.2023.111736
$Cr_{0.20}Fe_{0.71}Mn_{0.01}Ni_{0.08}$	0.89	$\mathbf{I_{corr}}$<br>$\log(\mathbf{I_{corr}})$	3.10×10^{-4}<br>-3.51	mA/cm^2<br>-	10.1016/j.corsci.2023.111302
**Flagged Alloys (Excellent Corrosion Resistance, Incomplete Sustainability Data)**					
$Zr_{0.43}Ti_{0.14}Cu_{0.14}Ni_{0.14}B$<br>*(Missing: B)*	N/A	$\mathbf{I_{corr}}$<br>$\log(\mathbf{I_{corr}})$	2.80×10^{-5}<br>-4.55	mA/cm^2<br>-	10.1016/j.mseb.2023.116267
$Zr_{0.47}Ti_{0.13}Cu_{0.13}Ni_{0.13}B$<br>*(Missing: B)*	N/A	$\mathbf{I_{corr}}$<br>$\log(\mathbf{I_{corr}})$	3.02×10^{-5}<br>-4.52	mA/cm^2<br>-	10.1016/j.mseb.2023.116267
$Zr_{0.38}Ti_{0.15}Cu_{0.15}Ni_{0.15}B$<br>*(Missing: B)*	N/A	$\mathbf{I_{corr}}$<br>$\log(\mathbf{I_{corr}})$	3.19×10^{-5}<br>-4.50	mA/cm^2<br>-	10.1016/j.mseb.2023.116267
$Zr_{0.27}Ti_{0.18}Cu_{0.18}Ni_{0.18}B$<br>*(Missing: B)*	N/A	$\mathbf{I_{corr}}$<br>$\log(\mathbf{I_{corr}})$	4.05×10^{-5}<br>-4.39	mA/cm^2<br>-	10.1016/j.mseb.2023.116267
$Zr_{0.33}Ti_{0.17}Cu_{0.17}Ni_{0.17}B$<br>*(Missing: B)*	N/A	$\mathbf{I_{corr}}$<br>$\log(\mathbf{I_{corr}})$	7.26×10^{-5}<br>-4.14	mA/cm^2<br>-	10.1016/j.mseb.2023.116267
**Industrial Benchmarks**					
C-276	0.84	$\mathbf{I_{corr}}$<br>$\log(\mathbf{I_{corr}})$	8.00×10^{-5}<br>-4.10	mA/cm^2<br>-	10.1016/j.phpro.2013.03.048
Ti-6Al-4V	0.88	$\mathbf{I_{corr}}$<br>$\log(\mathbf{I_{corr}})$	2.42×10^{-4}<br>-3.61	mA/cm^2<br>-	10.3390/ma17205026
Inconel 625	0.84	$\mathbf{I_{corr}}$<br>$\log(\mathbf{I_{corr}})$	3.8×10^{-4}<br>-3.42	mA/cm^2<br>-	10.1088/1742-6596/1948/1/012127
AL-6XN	0.87	$\mathbf{I_{corr}}$<br>$\log(\mathbf{I_{corr}})$	3.00×10^{-4}<br>-3.48	mA/cm^2<br>-	10.1088/2053-1591/ac1b64
SS 316L	0.89	$\mathbf{I_{corr}}$<br>$\log(\mathbf{I_{corr}})$	1.70×10^{-3}<br>-2.77	mA/cm^2<br>-	10.3390/met11040597
Nitinol 60	0.86	$\mathbf{I_{corr}}$<br>$\log(\mathbf{I_{corr}})$	5.57×10^{-4}<br>-3.25	mA/cm^2<br>-	www.sti.nasa.gov:NASA/CR—2016–218220
SS 304	0.89	$\mathbf{I_{corr}}$<br>$\log(\mathbf{I_{corr}})$	5.12×10^{-3}<br>-2.29	mA/cm^2<br>-	10.20964/2022.04.29

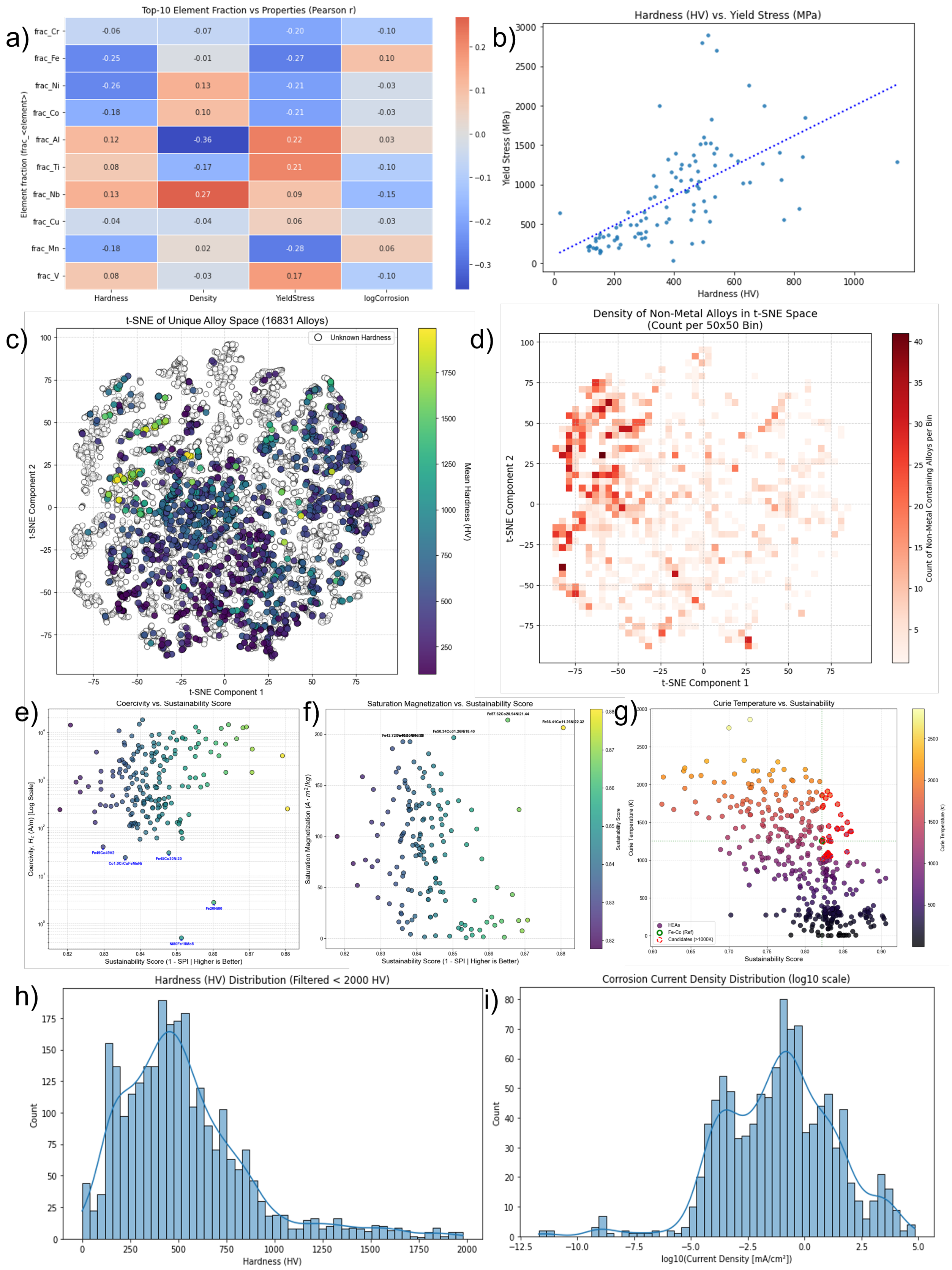

Figure S2: Trend analysis using DB2. a) Property-element correlations for hardness, density, yield strength and corrosion current density. b) Hardness-yield strength correlation. t-SNE visualizations showing higher c) hardness in regions with high d) count of non-metals. Other examples of sustainability maps for e) coercivity, f) saturation magnetization, and g) Curie temperature. Distribution of h) hardness and i) corrosion current density records available in DB2.
